# Epidemiology of Functional Diarrhea and Comparison with Diarrhea-Predominant Irritable Bowel Syndrome: A Population-Based Survey in China

**DOI:** 10.1371/journal.pone.0043749

**Published:** 2012-08-24

**Authors:** Yan-Fang Zhao, Xiao-Jing Guo, Zhan-Sai Zhang, Xiu-Qiang Ma, Rui Wang, Xiao-Yan Yan, Jia He

**Affiliations:** 1 Department of Health Statistics, Second Military Medical University, Shanghai, China; 2 Testing Center for Occupational Health, Shanghai Institute of Occupational Safety and Health, Shanghai, China; 3 Clinical Research Institute, Peking University, Beijing, China; The Australian National University, Australia

## Abstract

**Background:**

The epidemiology of functional diarrhea and its impacts on Chinese remain unclear, and there are no data on the comparative epidemiology of functional diarrhea and diarrhea-predominant irritable bowel syndrome (IBS-D). This study was to explore the epidemiology of functional diarrhea and its impacts, and to identify its distinction from IBS-D.

**Methods and Findings:**

A cross-sectional survey was conducted in 16078 respondents, who were interviewed under a randomized stratified multi-stage sampling design in five cities of China. All respondents completed the modified Rome II questionnaire, and the 36-item Short Form health survey (SF-36) was used for assessing health-related quality of life in 20% of the sample. Overall, 248 respondents (1.54%) had functional diarrhea and 277 (1.72%) had IBS-D. Functional diarrhea was positively associated with increasing age and body mass index (trend test *P*<0.05). The three most common symptoms for at least 3 weeks in the past months were loose, mushy or watery stools (n = 203, 81.85%), more than three bowel movements a day (n = 100, 40.32%) and having to rush to the toilet to have a bowel movement (n = 72, 29.03%). Meaningful impairment was observed in 5 of the 8 SF-36 domains in respondents with functional diarrhea. The demographics are mostly similar between the respondents with functional diarrhea and IBS-D; however, respondents with IBS-D had more frequent symptoms of diarrhea and even lower scores in SF-36 domains than those with functional diarrhea.

**Conclusions:**

The prevalence of functional diarrhea in China is substantially lower than that in Western countries and relatively higher than that in other Asian countries. It impaired health-related quality of life, and respondents with IBS-D have even worse quality of life. Further population-based studies are needed to investigate the epidemiology of functional diarrhea and the differences between functional diarrhea and IBS-D.

## Introduction

Chronic diarrhea is a common illness in Western countries, such as Australia, Canada, Ireland, and the United States [1]. It was reported that chronic diarrhea or loose or watery stools affected up to 26.9% of adults in the United States [2]. The presence of chronic diarrhea may affect patients’ quality of life, cause them to experience reduced productivity and increase the economic burden. [3] Available evidence indicated that chronic diarrhea represents a significant health care burden in the United States [3]. Individuals with diarrhea usually do not seek medical advice but treat themselves using over-the-counter remedies, by dietary modification or by doing nothing. Medical help is usually sought when diarrhea is more severe, is accompanied by fever or rectal bleeding, or results in prostration [4]. All these factors mean that chronic diarrhea is a major public health issue.

Diarrhea is termed chronic when it lasts for more than 4 weeks [5]. However, a universally accepted consensus definition has yet to be formulated. When underlying organic diseases are absent, a functional bowel disorder is thought to be present. The two major functional bowel disorders characterized by diarrhea are diarrhea-predominant irritable bowel syndrome (IBS-D) and functional diarrhea [6]. The Rome diagnostic criteria were developed as an appropriate standard to define diarrhea. According to Rome II, the primary factor that differentiates between these two diagnoses is that there is passage of loose or watery stools with abdominal pain or discomfort in patients with IBS-D.

It was reported that the prevalence of functional gastrointestinal disorders differed based on the geographical region and race [7], [8]. Several studies have been conducted to determine the prevalence of functional diarrhea in Western countries [9], [10]; however, there are limit data on epidemiology of functional diarrhea and its impacts from Asian countries to compare with Western countries. In Korean, the prevalence was only 0.8% [11]. Few population-based studies have estimated the prevalence of functional diarrhea in China. The epidemiology of functional diarrhea and its effects on Chinese people remain unclear. Furthermore, there are no data on the comparative epidemiology of functional diarrhea and IBS-D. Our study was conducted to investigate the epidemiology of functional diarrhea, as defined by Rome II, and its effects, and to determine how functional diarrhea is distinct from IBS-D in a large population sample of Chinese adults.

## Materials and Methods

As part of the large survey of the Systematic Investigation of Gastrointestinal Diseases in China (SILC), the main methods have been described in detail elsewhere [12], and are summarized here.

### Participants and Study Design

A total of 18 000 subjects aged 18–80 years were selected using a randomized, stratified, multi-stage sampling methodology in Shanghai, Beijing, Xi’an, Wuhan, and Guangzhou in China (3600 individuals for each region). All subjects were sampled from urban and rural residential areas in a 1∶1 ratio after stratification by the overall age and sex distribution for that region.

### Survey Instruments

All respondents completed a general information questionnaire, which included demographic information, including resident region, gender, age, sex, height, weight, education, total monthly family income, occupation, lifestyle habits (including smoking status, alcohol consumption and frequency of recreational exercise) and family history of gastrointestinal diseases.

A validated Chinese version of the modified Rome II questionnaire was used to determine the presence of functional diarrhea, IBS-D and other functional gastrointestinal disorders. The modified Rome II questionnaire used in our study included gastroduodenal, bowel and biliary items only. Functional diarrhea was defined in accordance with the Rome II criteria as loose, mushy, or watery stools, present more than three quarters of the time and no abdominal pain in the last 3 months [13]. Irritable bowel syndrome was defined according to Rome II as abdominal discomfort or pain that had two out of three features: relieved with defecation; onset associated with a change in frequency of stool; onset associated with a change in form (appearance) of stool. IBS was described as diarrhea-predominant (IBS-D) if patients had one or more of the following: more than three bowel movements a day; loose (mushy) or watery stools; urgency (having to rush to have a bowel movement) [14].

In addition, a random subsample of 20% of the total sample from each region was asked to complete the Chinese version of the 36-item Short Form health survey (SF-36), which measures health-related quality of life. Its reliability and validity have been tested [12], [15].

### Data Collection and Response Rate

The field work was conducted from April 2007 to January 2008. The sampled respondents completed the questionnaires by themselves, and trained and supervised facilitators are available to explain any questions that respondents were unclear. (Most questions were about the questionnaire formats, such as the skip rules in the Rome II questionnaire.) A total of 16 091 respondents completed the questionnaires with a response rate of 89.4%. Of them, 16 078 were suitable for analysis. The SF-36 and ESS questionnaires were completed by 3219 respondents (a response rate of 89.4%) and data from 3214 respondents were suitable for analysis. The demographics and lifestyle characteristics of the respondents have been described previously [12].

### Ethics Statements

The study was approved by the Ethics Committee of the Second Military Medical University, Shanghai, China. All respondents gave their informed consent to participate in the study and were free to discontinue their participation at any time.

### Statistical Analysis

SAS 9.1.3 software (SAS Institute, Cary, NC, USA) was used for data analyses. Odds ratios (ORs) and 95% confidence intervals (CIs) were calculated using multivariate logistic regression. The Cochran–Armitage test was used to detect trends. The chi-square test was used to compare the groups with functional diarrhea and with IBS-D and the *t*-test was used to compare the SF-36 between individuals with and without functional diarrhea or between individuals with functional diarrhea and those with IBS-D. All hypothesis tests used two-sided tests, and *P*-value of 0.05 or less was considered to be statistical significant.

## Results

### Prevalence, Demographics and Lifestyle Characteristics of Respondents with Functional Diarrhea

**Figure 1:**
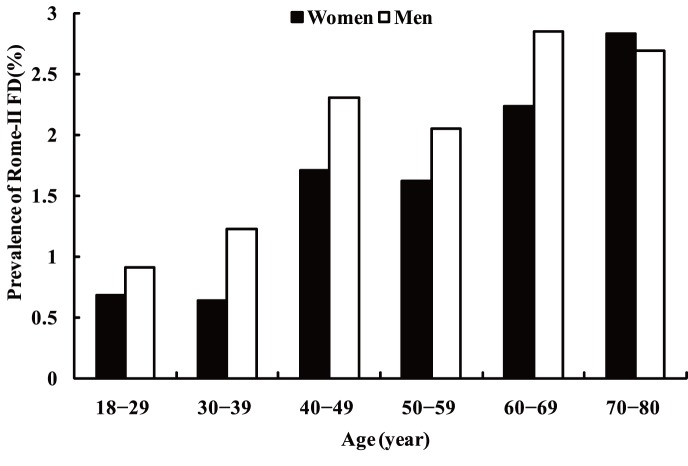
Age- and gender-specific prevalence of functional diarrhea based on a sample of 16078 respondents.

Of the 16078 respondents, a total of 248 respondents (1.54%; 95% CI: 1.35–1.73%) were classified as having functional diarrhea according to the Rome II criteria. The mean age of the respondents with functional diarrhea was 48.83 years. Functional diarrhea was more prevalent in men than in women without adjusting other factors (1.77% vs. 1.33%, *P* = 0.0257) and the prevalence of functional diarrhea increased with increasing age in both men and women (trend test: *P*<0.0001)(Figure 1). The prevalence of functional diarrhea in Shanghai, Beijing, Xi’an, Wuhan, and Guangzhou was 2.19%, 1.39%, 2.17%, 1.07% and 0.90%, respectively, and it varied significantly among the five study regions (*P*<0.001).

The demographic characteristics of respondents with and without functional diarrhea and the associated potential risk factors are presented in Table 1. On multivariate analysis, individuals aged 40–80 years, especially those aged 60–80 years, were significantly more likely to have functional diarrhea than those aged 18–29 years (60–69 years and 70–80 years vs. 18–29 years, OR: 2.63; 95% CI: 1.55–4.46 and OR: 2.95; 95% CI: 1.62–5.36, respectively). Respondents with higher body mass index (BMI) were more likely to report functional diarrhea than those whose BMI were lower than 18.5 kg/m^2^ (18.5–22.9 kg/m^2^, 23.0–27.9 kg/m^2^,≥28 kg/m^2^ vs. <18.5 kg/m^2^, OR: 2.36; 95% CI: 1.09–5.11, OR: 3.49; 95% CI: 1.61–7.58, and OR: 3.83; 95% CI: 1.63–8.99). Positive association was also significant between functional diarrhea and increasing BMI in trend test (*P* = 0.0147). Respondents with a family history of gastrointestinal disease were also more likely to report functional diarrhea (OR: 1.58; 95% CI: 1.08–2.31). No significant association was found between functional diarrhea and living environment, sex, education, occupation, total monthly family income, smoking status, alcohol consumption or frequency of recreational exercise.

**Table 1:** Characteristics of respondents with and without functional diarrhea and the associated potential risk factors.

Variables	With functional diarrhea (*n* = 248) *n* (%)	Without functional diarrhea (*n* = 15,830) *n* (%)	Multivariate OR (95% CI)
Environment			
Urban	127(51.21)	7945(50.19)	1.00
Rural	121(48.79)	7885(49.81)	0.94(0.71,1.25)
Sex			
Female	112(45.16)	8278(52.29)	0.82(0.58,1.15)
Male	136(54.84)	7552(47.71)	1.00
Age (years)			
18−29	29(11.69)	3651(23.06)	1.00
30−39	34(13.71)	3641(23.00)	1.04(0.63,1.73)
40−49	76(30.65)	3736(23.60)	2.10(1.33,3.32)
50−59	45(18.15)	2423(15.31)	1.87(1.13,3.40)
60−69	38(15.32)	1465(9.25)	2.63(1.55,4.46)
70−80	26(10.48)	914(5.77)	2.95(1.62,5.36)
BMI (kg/m^2^)^a^			
<18.5	7(2.82)	1473(9.35)	1.00
18.5–22.9	96(38.71)	7625(48.38)	2.36(1.09,5.11)
23.0–27.9	120(48.39)	5651(35.85)	3.49(1.61,7.58)
≥28	25(10.08)	1013(6.43)	3.83(1.63,8.99)
Education			
None/primary school	63(25.40)	3119(19.71)	1.00
Secondary/high school	143(57.66)	9787(61.83)	0.95(0.66,1.35)
College graduates or beyond	42(16.94)	2922(18.46)	1.15(0.66,1.98)
Occupation			
Office worker	64(25.81)	4148(26.24)	1.00
Manual worker	184(74.19)	11661(73.76)	0.84(0.59,1.20)
Total monthly family income (yuan)			
≤1999	148(59.92)	8667(54.89)	1.00
2000–4999	84(34.01)	5879(37.23)	0.81(0.60,1.09)
≥5000	15(6.07)	1244(7.88)	0.66(0.37,1.16)
Smoking status			
Never smoker	156(62.90)	11074(69.97)	1.00
Current smoker	80(32.26)	4351(27.49)	1.08(0.75,1.55)
Ex-smoker	12(4.84)	402(2.54)	1.27(0.66,2.42)
Alcohol consumption			
No	188(75.81)	12625(79.77)	1.00
Yes	60(24.19)	3202(20.23)	1.04(0.74,1.46)
Family history of GI diseases			
No	216(87.10)	14428(91.18)	1.00
Yes	32(12.90)	1395(8.82)	1.58(1.08,2.31)
Frequency of recreational exercise			
Never	40(16.13)	2091(13.24)	1.00
Less than weekly	12(4.84)	1351(8.55)	0.52(0.27,1.01)
At least weekly but less than daily	167(67.34)	2165(13.70)	0.74(0.46,1.22)
Daily	571(60.4)	10192(64.51)	0.80(0.56,1.14)

BMI, body mass index; OR, odds Ratio; CI, confidence interval; GI, gastrointestinal.

a BMI ranges are appropriate for the Asian population (underweight: <18.5 kg/m^2^; normal: 18.5–22.9 kg/m^2^; overweight: 23.0–27.9 kg/m^2^; obese: ≥28.0 kg/m^2^).

### Range of Symptoms in Respondents with Functional Diarrhea

**Figure 2:**
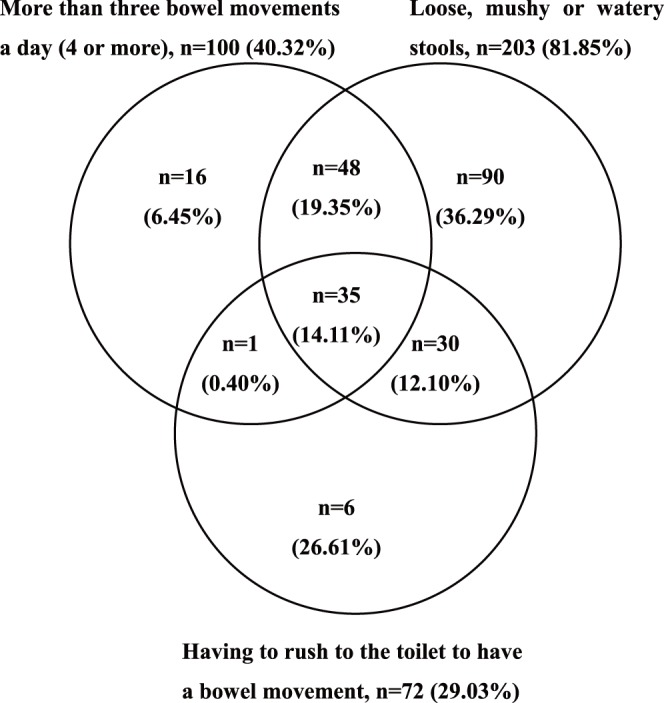
Overlap between the three most common symptoms reported by most respondents in the investigation (n = 248).

The respondents with functional diarrhea reported a wide range of symptoms of diarrhea for at least 3 weeks in the past months, and many reported more than one symptom. The three most common symptoms were loose, mushy or watery stools (n = 203, 81.85%), more than three bowel movements a day (n = 100, 40.32%) and having to rush to the toilet to have a bowel movement (n = 72, 29.03%). Figure 2 shows the overlap of these three symptoms. Other reported symptoms included feeling of incomplete emptying after a bowel movement (n = 63, 25.40%), abdominal fullness, bloating or swelling (n = 53, 21.37%) and passing mucus (slime) during a bowel movement (n = 24, 9.68%). A total of 81 functional diarrhea respondents (32.66%) reported that they had at least three of the above symptoms.

### Burden of Functional Diarrhea

**Figure 3:**
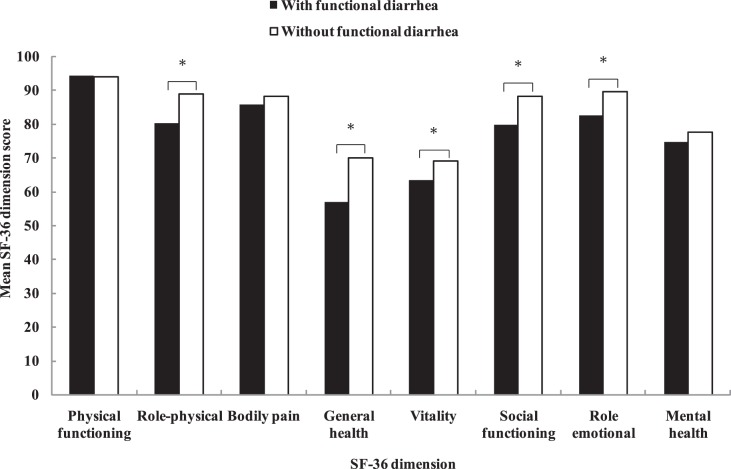
Health-related quality of life in individuals with and without functional diarrhea.

Respondents with functional diarrhea had significantly lower scores in role-physical, general health, vitality, social functioning, and role-emotional domains of the SF-36 than those without (all *P*<0.05) (Figure 3). The most substantial difference was observed in the general health domain. For respondents with functional diarrhea, there were no significant differences in SF-36 domain scores between men and women. The scores of role-physical domain and social functioning domain differed between respondents with fewer symptoms of functional diarrhea and those with three or more symptoms (*P*<0.05). However, there were no significant differences in other domain scores (data not shown).

### Comparison between Respondents with Functional Diarrhea and those with IBS-D

Among the total of respondents, 277 (1.72%) had IBS-D. Respondents with functional diarrhea had higher BMI on average than those with IBS-D (23.94 kg/m^2^ vs. 23.17 kg/m^2^, *P* = 0.0112). A family history of gastrointestinal disease was more common in respondents with IBS-D than in those with functional diarrhea (20.58% vs. 12.90%, *P* = 0.0193). Respondents with functional diarrhea and those with IBS-D had similar demographic characteristics with regard to environment, sex, education, occupation, total monthly family income, smoking status, alcohol consumption and frequency of recreational exercise (data not shown).

Table 2 shows the differences in various symptoms of diarrhea between the respondents with functional diarrhea and those with IBS-D. The symptoms, including more than three bowel movements a day, having to rush to the toilet to have a bowel movement, feeling of incomplete emptying after a bowel movement, passing mucus (slime) during a bowel movement, and abdominal fullness, bloating or swelling, were more frequent in respondents with IBS-D than in those with functional diarrhea (all *P*<0.05 ). The symptom of loose, mushy or watery stools was similar in respondents with functional diarrhea and those with IBS-D.

**Table 2:** The differences of various diarrhea symptoms between respondents with functional diarrhea and with diarrhea-predominant irritable bowel syndrome (IBS-D).

Bowel disorders	Functional diarrhea (n = 248)	IBS-D (n = 277)	*P* value
More than three bowel movements a day (4 or more) [n (%)]	100 (40.32)	147 (53.07)	0.0035
Loose, mushy or watery stools [n (%)]	203 (81.85)	226 (81.59)	0.9372
Having to rush to the toilet to have a bowel movement [n (%)]	72(29.03)	156 (56.32)	<0.0001
Feeling of incomplete emptying after a bowel movement [n (%)]	63 (25.40)	94 (33.94)	0.0332
Passing mucus (slime) during a bowel movement [n (%)]	24 (9.68)	55 (19.86)	0.0011
Abdominal fullness, bloating or swelling [n (%)]	53 (21.37)	137 (49.46)	<0.0001

**Figure 4:**
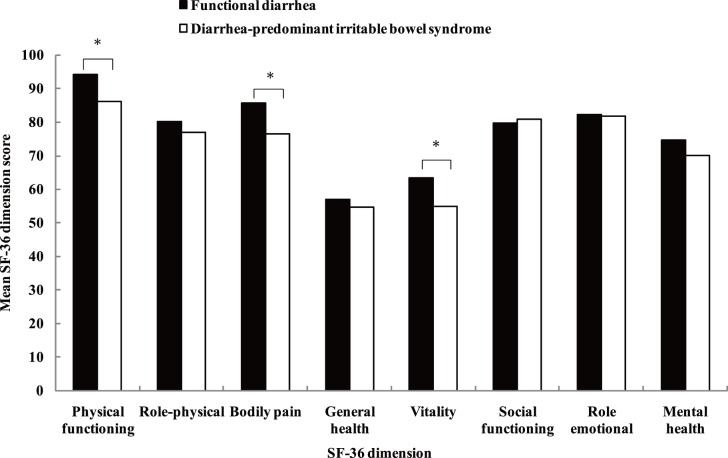
Health-related quality of life in individuals with functional diarrhea and with diarrhea-predominant irritable bowel syndrome.

The comparison of health-related quality of life between respondents with functional diarrhea and those with IBS-D is shown in Figure 4. Respondents with functional diarrhea had higher scores than those with IBS-D in the physical functioning, bodily pain and vitality domains (all *P*<0.05); however, no significant difference was found in the other SF-36 domains.

## Discussion

The prevalence of functional diarrhea and its impacts on people’s health have received little systematic investigation in the general population in Asia. In this large, population-based epidemiological study, a randomized stratified multi-stage sampling method was used, and a total of 16 078 respondents from five regions in China were investigated. The overall prevalence of functional diarrhea as defined by Rome II was 1.54%.

The different study designs and definitions of functional diarrhea or chronic diarrhea in various studies have provided researchers with diverse prevalence rates, which made international comparisons difficult. A study in a Swedish adult population reported that the prevalence of self-reported diarrhea was 9.8% [17]. A study compared the prevalence of diarrhea in the community in Australia, Canada, Ireland and the United States, and showed that the prevalence was 6.4%, 7.6%, 3.4% and 7.6%, in which diarrhea was defined as more than 3 loose stools or bowel movements in any 24 hours period in the four weeks prior to interview [1]. A population-based survey in Canada using Rome II criteria reported that the prevalence was 8.5% [9].A survey among healthy volunteers in Mexico City using the Rome II criteria indicated that the prevalence of functional diarrhea was 3.4% [16]. Compared with Western countries, the prevalence of functional diarrhea in China was substantially lower. However, compared with other countries in Asia, the prevalence was relatively higher. For example, a large sample (n = 18180) of the general population study in Iran reported that only 0.2% had functional diarrhea according to the Rome III criteria [6]. A survey using Rome II in randomly selected residents in Korea showed the prevalence of diarrhea was 0.8% [11]. In our study, the prevalence of functional diarrhea was even higher in Shanghai and Xi’an. Different dietary habits and demographic variability in this vast country of China might have caused the significant difference, as in other studies [18].

Our study showed that the prevalence of functional diarrhea was slightly higher in men than in women, but the difference was not significant after adjusting other factors, and this is consist with other studies [7], [9], [11]. Some studies reported that the prevalence of functional diarrhea was higher in men than in women [16], [19]. It might be that men had a faster colonic transit time than women, and women had delayed gastric empting of liquids and solids compared to that in men [20], [21].

A previous study reported that the prevalence of functional diarrhea was positively associated with age [6], which is consistent with our study. We found that BMI was positively associated with functional diarrhea. This may be that BMI is correlated inversely with colonic transit time and overweight/obese patients have more severe symptoms of urgency, loose stools and more stools per day [22]. It was reported that higher BMI showed an independent and significant relationship to faster colonic transit [23]. Our study also found that functional diarrhea was significantly associated with a family history of gastrointestinal diseases. Common demographic risk factors and a genetic predisposition may play a role in this relationship. Education, occupation, family income, smoking status, alcohol consumption and frequency of recreational exercise were not associated with functional diarrhea in our study. However, there are no adequate data on these issues to be used to compare the results with other countries. Some studies on functional diarrhea defined by Rome II did not report adequate data, and different methodologies in different studies don’t allow direct comparisons [1]. These indicate that more population-based studies are needed to clarify these issues.

Demographics of respondents with functional diarrhea and those with IBS-D were similar in our study. The primary difference is that respondents with functional diarrhea had higher BMI on average than those with IBS-D. We have previously reported that BMI was not associated with IBS [14], and this may explain the difference.

It was reported that patients with diarrhea had a wide variety of symptoms [24]. The symptom of loose, mushy or watery stools was the most frequent symptom of functional diarrhea. In fact, most people apply the term diarrhea to loose or watery stools. It was indicated that stool form, not frequency, defined diarrhea, and how often a symptom must occur to be significant depends on its troublesomeness [25]. According to the Rome criteria, the primary difference in symptoms between functional diarrhea and IBS-D is that abdominal pain must be present in the latter. A study in Iran using Rome III, reported that some symptoms were more frequent in IBS-D than functional diarrhea [6]. In our study, IBS-D also had more frequency of the diarrhea symptoms, such as more than three bowel movements a day, having to rush to the toilet to have a bowel movement, feeling of incomplete emptying after a bowel movement, and so on.

Having diarrheal illness was associated with a high rate of physician visits and work absenteeism, and with heavy social and economic costs [3], [7], [26]. However, there have been very few studies evaluating the impacts of functional diarrhea on health-related quality of life in the general population. In the present study, we observed that the health-related quality of life was significantly impaired in respondents with functional diarrhea. Comparison with functional diarrhea, respondents with IBS-D had even worse health-related quality of life. This may be due to that people with IBS-D had more frequent symptoms [6]. Further studies are required to explore the quality of life of functional diarrhea and its comparison with IBS-D.

Our study had several strengths. It is the largest population-based epidemiological survey of functional diarrhea ever conducted in China, which spans five major population centers, using global consensus-based definition of functional diarrhea [27]. This study provided representative, high-quality and generalizable data on the prevalence of functional diarrhea in China, which used a validated survey methodology and achieved a high response rate that minimized the potential for responder bias. Moreover, this study involved a comparison between functional diarrhea and IBS-D, which has the potential to make a major contribution to the epidemiological understanding of functional diarrhea and IBS-D. Admittedly, with the cross-sectional natural, the directionality of any association can’t be assessed. Another limitation is that medication history was not systematically recorded. Besides, patients with organic caused of diarrhea were not excluded in the study.

### Conclusions

In conclusion, this large-scale population-based study found that the prevalence of the Rome II defined functional diarrhea in China was substantially lower than that in Western countries and relatively higher than other Asian countries. Functional diarrhea impaired health-related quality of life in Chinese adults. The demographics were mostly similar between functional diarrhea and IBS-D; however, respondents with IBS-D have more frequent clinical symptoms and worse health-related quality of life. Further population-based studies are needed to investigate the epidemiology of functional diarrhea and the differences between functional diarrhea and IBS-D.
